# P-875. *Bacteroides* spp. drug resistance in patients with anaerobic bacteremia: a multicentric retrospective observational study

**DOI:** 10.1093/ofid/ofae631.1066

**Published:** 2025-01-29

**Authors:** Ryotaro Kubo, Naoki Iwanaga, Kentaro Nagaoka, Masataka Yoshida, Kazuaki Takeda, Shotaro Ide, Takahiro Takazono, Kosuke Kosai, Yoshitomo Morinaga, Koichi Izumikawa, Yoshihiro Yamamoto, Katsunori Yanagihara, Shigeru Kohno, Hiroshi Mukae

**Affiliations:** Nagasaki University Hospital, Nagasaki, Nagasaki, Japan; Nagasaki University Hospital, Nagasaki, Nagasaki, Japan; Toyama University Graduate School of Medicine and Pharmaceutical Sciences, Toyama, Toyama, Japan; Nagasaki University Hospital, Nagasaki, Nagasaki, Japan; Nagasaki University Hospital, Nagasaki, Nagasaki, Japan; Nagasaki University Hospital, Nagasaki, Nagasaki, Japan; Nagasaki University Graduate School of Biomedical Sciences, Nagasaki, Nagasaki, Japan; Nagasaki University, Nagasaki, Nagasaki, Japan; Toyama University Graduate School of Medicine and Pharmaceutical Sciences, Toyama, Toyama, Japan; Nagasaki University, Nagasaki, Nagasaki, Japan; Toyama University Graduate School of Medicine and Pharmaceutical Sciences, Toyama, Toyama, Japan; Nagasaki University, Nagasaki, Nagasaki, Japan; Nagasaki University, Nagasaki, Nagasaki, Japan; Nagasaki University, Nagasaki, Nagasaki, Japan

## Abstract

**Background:**

*Bacteroides* spp. are important causative anaerobes in intra-abdominal infections; however, recent reports have shown a trend toward drug resistance in non-*Bacteroides fragilis*. The present study aimed to evaluate bacteremia due to *Bacteroides* spp. by performing a multicentric retrospective observational study.

**Methods:**

Obligate anaerobes isolated from blood culture specimens were collected from patients at Nagasaki University Hospital and five core hospitals in Toyama Prefecture from January 2012 to March 2022. Of a total of 528 patients, 102 specimens with *B. fragilis* and 72 with non-*B. fragilis* were clinically characterized using Fisher’s exact test and multiple logistic regression analysis. Drug-resistant rates over time were analyzed by the Spearman rank correlation test.

**Results:**

In the non-*B. fragilis* group, compared to the *B. fragilis* group, the clindamycin resistance rate (non-*B. fragilis* group [59.7%] vs. *B. fragilis* group [38.2%] odds ratio [OR]=2.40 [95% confidence interval [CI]; 1.23–4.43] p=0.0058) and carbapenem resistance rate (non-*B. fragilis* group [27.8%] vs. *B. fragilis* group [10.8%] OR=3.18 [95%CI; 1.41–6.78] p=0.0049) was significantly increased. Furthermore, in the non-*B. fragilis* group, excluding patients who died owing to an underlying disease, a significantly increased 30-d mortality rate (non-*B. fragilis* group [16.7%] vs. *B. fragilis* group [5.9%] OR=3.20 [95%CI; 1.15–8.91] p=0.0251) was observed. Moreover, as of risk factors for 30-d mortality in non-*B. fragilis* bacteremia, SOFA score (adjusted OR=1.30 [95%CI; 1.11–1.57] p=0.0027) and carbapenem resistance (adjusted OR=7.05 [95%CI; 1.54–41.9] p=0.0171) were elucidated. Of note, in the non-*B. fragilis* group, rather than the *B. fragilis* group, the carbapenem resistance rate (non-*B. fragilis* group [r = 0.77, p=0.0141] vs. *B. fragilis* group [r = 0.17, p=0.6433]) and tazobactam/piperacillin resistance rate (non-*B. fragilis* group [r = 0.78, p=0.0120] vs. *B. fragilis* group [r = -0.05, p=0.8988]) have increased over time.

**Conclusion:**

Non-*B. fragilis* isolated from blood cultures, compared to *B. fragilis*, exhibited an increasing trend in carbapenem and tazobactam/piperacillin resistance and contributed to 30-d mortality.

**Disclosures:**

**Shigeru Kohno, n/a**, SHIONOGI & CO., LTD.: Board Member|SHIONOGI & CO., LTD.: Grant/Research Support **Hiroshi Mukae, M.D., Ph.D.**, AstraZeneca: Lecture fees|Gilead Sciences: Lecture fees|GSK: Lecture fees|MSD: Advisor/Consultant|MSD: Lecture fees|Pfizer: Lecture fees|Shionogi & Co., Ltd.: Advisor/Consultant|Shionogi & Co., Ltd.: Grant/Research Support|Shionogi & Co., Ltd.: Lecture fees

